# Experiences During the COVID-19 Pandemic: A Survey of Biosafety Professionals

**DOI:** 10.1089/apb.2022.0012

**Published:** 2022-09-14

**Authors:** David Gillum, Henry Wyneken, Jennifer Fletcher, Karl Nubbe, Kathleen M. Vogel

**Affiliations:** ^1^ Environmental Health and Safety, Arizona State University, Tempe, Arizona, USA.; ^2^ Gryphon Scientific, Takoma Park, Maryland, USA.; ^3^ Accountability, Assessment and Research Department, Chandler Unified School District, Chandler, Arizona, USA.; ^4^ School for the Future of Innovation and Society, Arizona State University, Tempe, Arizona, USA.

**Keywords:** SARS-CoV-2, COVID-19, biosafety, survey, pandemic, stress

## Abstract

**Introduction:**

Biosafety professionals were called to action during the COVID-19 pandemic. They were tasked with prescribing measures to keep workers and the community safe while often not having accurate information at their fingertips. Understanding biosafety professionals' experiences may help shape new approaches that could further advance preparedness and resilience goals for future pandemics. This article discusses the overall response efforts of the biosafety community.

**Objectives:**

The main objective of this article is to quantitatively and qualitatively interrogate the responses to an email survey sent to individuals with biosafety responsibilities during the COVID-19 pandemic. This article catalogues those responses and the different aspects in how biosafety professionals were involved in the pandemic. The focus of this research was on aggregate data and summarized results.

**Results:**

A total of 609 out of 654 respondents fully completed the survey, equating to a 93.1% completion rate. Respondents were individuals with varying levels of COVID-19–related responsibilities participating in emergency preparedness and planning, developing laboratory diagnostic capabilities, reviewing clinical trials, developing safety guidelines, writing return-to-work and quarantine procedures, and participating in press releases and communications.

**Conclusions:**

Biosafety professionals played important roles during the COVID-19 pandemic, from developing safety protocols for laboratories to resourcing personal protective equipment during a global shortage. They experienced challenges when balancing their home/work lives. Some biosafety professionals were very involved in clinical trials and vaccination efforts, but most were not. Overall, there were significant differences in how biosafety professionals were involved in pandemic response efforts.

## Introduction

Biosafety professionals faced many challenges during the COVID-19 pandemic.^1,2^ They were required to manage facets of protecting individuals within their respective organizations and client base, and within their own communities.^2–4^ Biosafety professionals were asked to identify effective cleaning products and disinfectants,^5^ resource personal protective equipment (PPE),^6^ develop novel respirator reuse strategies,^7^ provide guidance to clinical and research laboratories,^8^ justify whether plexiglass or other engineering controls were effective,^9^ evaluate air filtration systems,^10^ develop worker safety training modules,^3^ and assess work environments to better protect workers against SARS-CoV-2,^11^ long before reliable scientific data were available. This was often accomplished using common sense as well as well-established biosafety risk assessment methodologies.^12,13^

Understanding biosafety professionals' experiences during the COVID-19 pandemic can help shape novel approaches and best practices for emergency preparedness and resiliency planning to help prevent and respond to future epidemic and pandemic disease outbreaks.^14^ In November 2021, researchers at Arizona State University conducted a study to assess the factors and conditions impacting biosafety professionals during the global outbreak. The goal of the survey was to better understand the unique ways that the biosafety profession participated in the response. This article uses qualitative and quantitative analyses to summarize and provide insight into the vital role played by biosafety professionals during the 2020–2021 COVID-19 pandemic.

## Methods

### Procedure

An email survey (see Supplementary Data S1 for survey questions) of biosafety professionals was conducted between November 16, 2021 and November 26, 2021. In total, 654 individuals affiliated with the ABSA International, and individuals listed as Institutional Biosafety Committee (IBC) contacts in accordance with the National Institutes of Health Office of Science Policy, were sent a 25-question survey. Two respondents did not respond to the over 18 years of age question, therefore, they were not offered the survey.

Forty-one respondents said they were not employed in a position with biosafety responsibilities and four did not respond to the question; these individuals were also not offered the survey. The remaining 609 respondents fully completed the survey, equating to a 93.1% completion rate. Participation in the survey was optional and voluntary. Because respondents were not required to complete every question, the total number of responses for each question varied. The focus of this research was on aggregate data and summarized results. All research complied with relevant Federal guidelines and institutional policies related to research on human subjects. The institutional review board (IRB) at Arizona State University (IRB No. 00014883) approved the research.

### Respondents

There were a total of 609 survey respondents of whom 383 (62.9%) worked in the United States (see Table 1 for a list of participating countries and Table 2 for descriptive details on each question). The respondents worked in the following sectors: academic (47.9%, *n* = 292), government (24.3%, *n* = 148), commercial (11.0%, *n* = 67), nonprofit (6.2%, *n* = 38), and other (3.4%, *n* = 21). Respondents varied in place of employment: laboratory (52.2%, *n* = 318), other (14.8%, *n* = 90), healthcare (12.6%, *n* = 77), consulting (6.9%, *n* = 42), pharmaceutical (3.3%, *n* = 20), and manufacturing (2.3%, *n* = 14).

**Table 1.** List of countries

	*n*	Percent
Afghanistan	1	0.2
Argentina	3	0.5
Armenia	1	0.2
Australia	2	0.4
Austria	1	0.2
Bangladesh	1	0.2
Barbados	1	0.2
Belgium	1	0.2
Bolivia	1	0.2
Brazil	3	0.5
Canada	23	4.1
Chile	3	0.5
China	2	0.4
Costa Rica	1	0.2
Egypt	8	1.4
Ethiopia	4	0.7
France	1	0.2
Gabon	1	0.2
Georgia	2	0.4
Germany	2	0.4
Greece	1	0.2
Hong Kong (S.A.R.)	1	0.2
India	5	0.9
Indonesia	2	0.4
Iraq	4	0.7
Italy	1	0.2
Japan	1	0.2
Jordan	3	0.5
Kenya	5	0.9
Kuwait	1	0.2
Lebanon	1	0.2
Libyan Arab Jamahiriya	1	0.2
Mauritius	1	0.2
Mexico	5	0.9
Micronesia, Federated States of…	1	0.2
Morocco	3	0.5
Netherlands	4	0.7
New Zealand	3	0.5
Nigeria	13	2.3
Pakistan	11	2.0
Peru	3	0.5
Philippines	6	1.1
Poland	1	0.2
Qatar	1	0.2
Russian Federation	1	0.2
Saudi Arabia	4	0.7
Sierra Leone	1	0.2
Singapore	4	0.7
Slovenia	1	0.2
South Africa	2	0.4
Spain	1	0.2
Sweden	1	0.2
Switzerland	2	0.4
Thailand	1	0.2
Trinidad and Tobago	1	0.2
Tunisia	2	0.4
Turkey	1	0.2
Uganda	2	0.4
United Arab Emirates	1	0.2
United Kingdom of Great Britain and Northern Ireland	3	0.5
United Republic of Tanzania	2	0.4
United States of America	383	69.0
Yemen	2	0.4

**Table 2.** Descriptive statistics

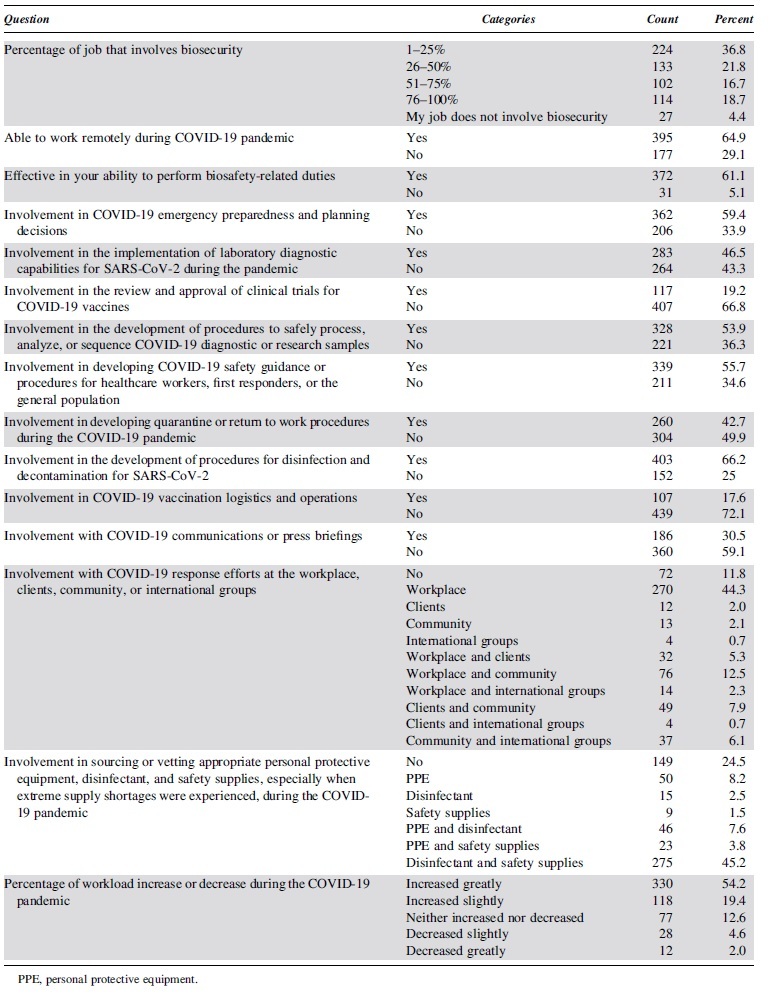

### Measures

The survey questions were designed to gain insights into the activities biosafety professionals did during COVID-19 while collecting demographic information—industry, sector, and location. Specifically, we were interested in understanding whether respondents were involved in remote work during the pandemic and their effectiveness working remotely and addressing the following topics related to COVID-19: response efforts, emergency preparedness, laboratory diagnostic capabilities, review and approval of vaccine clinical trials, diagnostic and research sample process/analysis, safety guidance, sourcing and vetting of PPE or supplies, quarantine and return-to-work procedures, disinfection and decontamination procedures, vaccine delivery logistics and operations, and communications and press briefings.

### Data Analysis

The survey included both quantitative and qualitative questions, with analysis conducted independently so the findings from the qualitative study did not influence the quantitative analysis and vice versa. Quantitative analysis began by using only respondents who responded yes to being of age 18 years or older, currently employed in a position with biosafety responsibilities, and responses to questions did not include “Not Applicable” or “Unknown.” Univariate analysis using a *p* value of 0.05 was conducted using chi square tests, *t*-tests, and analysis of variance to determine whether significant differences existed. Given the significant differences found in prior biosafety articles^15–18^ between location, place of employment, and sector, the univariate analysis anticipated finding similar differences.

Next, predictive analytics was conducted because the univariate differences found in the statistical analysis were less insightful than desired. Predictive analytics are valuable to learn more about the data that researchers may not have considered. Given the unique context of the pandemic, predictive analytics can provide a new perspective on trends or patterns that may have occurred. As such, multivariate analysis occurred by entering all independent (e.g., workload change, biosecurity level, working remote, place of employment, sector, and location) variables and dependent variables (e.g., questions specific to COVID-19 involvement) to search for possible clusters. Then models were created using R software.^19^

The method^20^ utilized the application of Tukey's range test^21^ to logistic regression models, which was implemented with the *glht* function from the R package *multcomp.*^19^ Specific contrasts were estimated in a logistic regression model and displayed using the R packages *ggplot2*^22^ and *ggthemes.*^23^ Certain components were included as covariates in the model (i.e., biosecurity responsibility level, change in workload, location, sector, and industry). We could not establish significant findings with an ordinal logistic regression model using the *polr* function from the *MASS* package.^24^ Respondents who did not give their location, sector, or industry were excluded from the multivariate analysis—these respondents tended to have much lower response rates on the individual questions. The multivariate analysis did not follow a preregistered strategy.

Three criteria were used to justify the predictive analytics model and its findings: differences are explainable, differences are severely tested, and the magnitude of the findings is large enough. It is expected that findings are intuitive. More importantly, though, to guard against bias in the findings, all contrasts that meet data-based thresholds (i.e., *p* values less than X or coefficients with magnitude greater than Y) are listed in Supplementary Data S2. Second, the findings must stand up to severe tests.^25^

**Figure 1.**
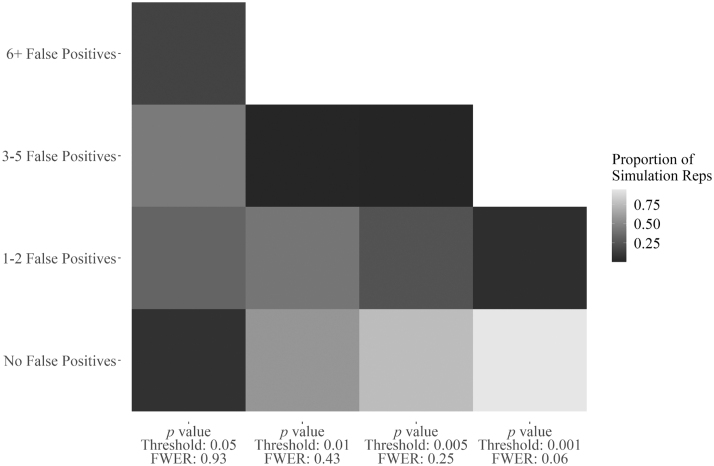
Explanation for use of *p* value threshold of 0.001. Risk of false positives across simulations of the null data when all pairwise comparisons are considered with a locally Tukey's HSD FWER-adjusted *p* value. FWER, family wise error rate; HSD, honest significant difference.

Reported *p* values for the multivariate main effect findings, which are <0.001, are based on an adjusted *p* value that locally controls the family wise error rate (FWER) among all pairwise comparisons for that question and factor (see Supplementary Data S2 for more details regarding the statistical analysis). Figure 1 displays what happens if *p* = 0.05 were used as a significance cutoff. The global FWER in this scenario is 93% compared with 6% when using a more conservative significance level of 0.001.

The data were searched for meaningful differences, and using a more difficult threshold was part of an attempt to make this an “honest hunting” expedition.^26^ This makes the method and results reliable but does mean some findings could be true despite not meeting the established significance level. As such, additional descriptive statistics and qualitative findings are presented. Third, effect sizes must be meaningful. All reported claims represent differences in the probability of answering yes, with magnitudes of at least 10%. Odds ratios (ORs) are used to report effect sizes—calculated from the models already described, not from the raw proportions in the data.^27^ Please refer to the Supplementary Data S2 for details about the method.

Finally, qualitative analysis was used to analyze two open-ended questions asking respondents to describe what challenges they faced during the pandemic. The responses of two questions were merged due to the similarity of the questions and responses received. Responses were analyzed using Charmaz's approach to grounded theory.^28^ Initial line-by-line coding asking, “what is happening,” by reading through all the data occurred first. In a second read through first level, descriptive codes were created. To ensure there was no bias in the data analysis and to acquire triangulation,^29^ three colleagues were invited to review the data for themes, and any variation was handled through discussion to achieve consensus.

## Results

### Descriptive Statistics

**Figure 2.**
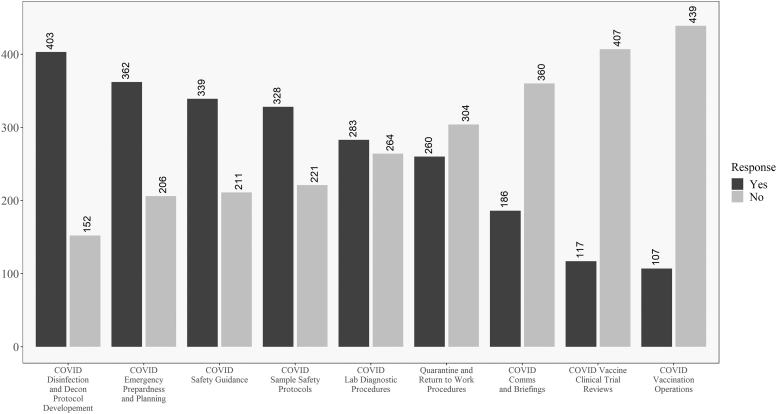
Descriptive results for all respondents on the COVID-19 involvement questions. Questions ordered from highest to lowest of “yes” responses.

Figure 2 shows how respondents responded to the COVID-19 involvement questions in order from the most “yes” responses to the most “no” responses. This chart shows that for most respondents, they were performing traditional biosafety functions during the pandemic. This included the development of SARS-CoV-2 disinfection and decontamination procedures, COVID-19 safety guidelines, and diagnostic laboratory safety procedures. Strikingly, many biosafety professionals were not involved in the creation of return-to-work and quarantine procedures, COVID-19 communications and briefings, SARS-CoV-2 clinical trials, and vaccination endeavors.

**Figure 3.**
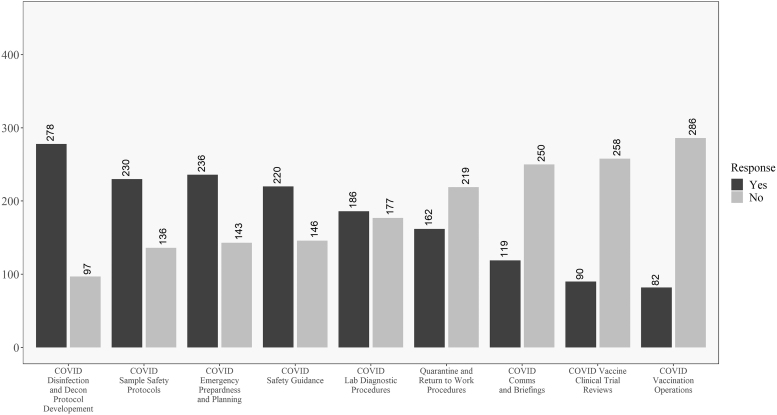
Descriptive results for respondents in the United States on the COVID-19 involvement questions. Questions ordered from highest to lowest of “yes” responses.

**Figure 4.**
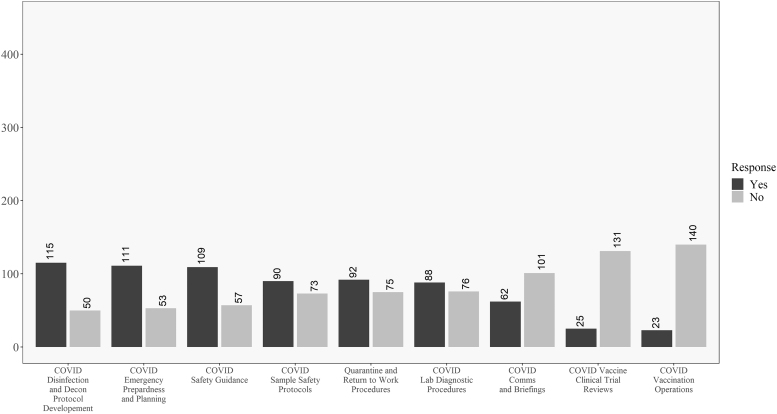
Descriptive results for respondents outside the United States on the COVID-19 involvement questions. Questions ordered from highest to lowest of “yes” responses.

In addition, Figures 3 and 4 reveal how these responses change when looking at the data for respondents within the United States versus those outside the United States. One difference of note is that respondents in the United States were less involved in developing return-to-work and quarantine procedures than their non U.S. counterparts. In opposition to this, U.S. respondents were more involved in vaccine review and operations and less involved in communications and briefings, as compared to non U.S. respondents. Significant differences between the groups will be explored in the univariate analysis where sample size is taken into account.

**Figure 5.**
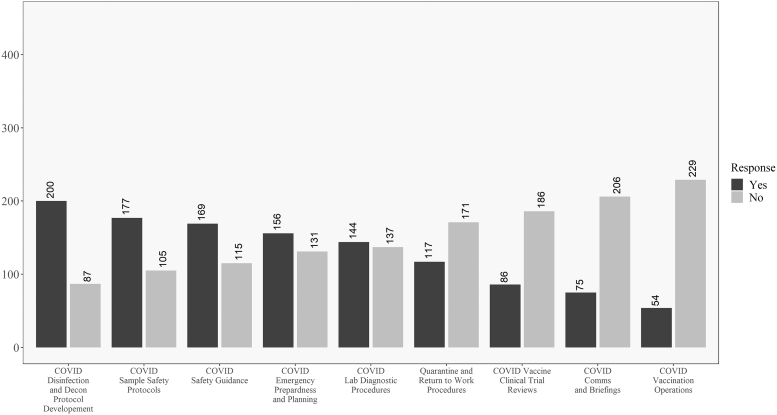
Descriptive results for academic respondents on the COVID-19 involvement questions. Questions ordered from highest to lowest of “yes” responses.

**Figure 6.**
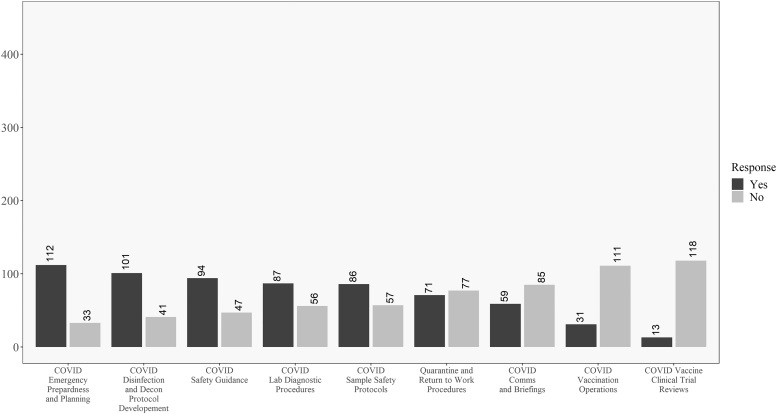
Descriptive results for government respondents on the COVID-19 involvement questions. Questions ordered from highest to lowest of “yes” responses.

**Figure 7.**
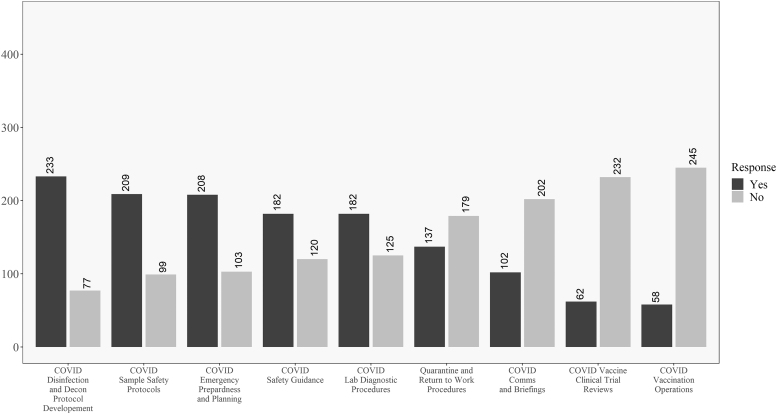
Descriptive results for laboratory respondents on the COVID-19 involvement questions. Questions ordered from highest to lowest of “yes” responses.

**Figure 8.**
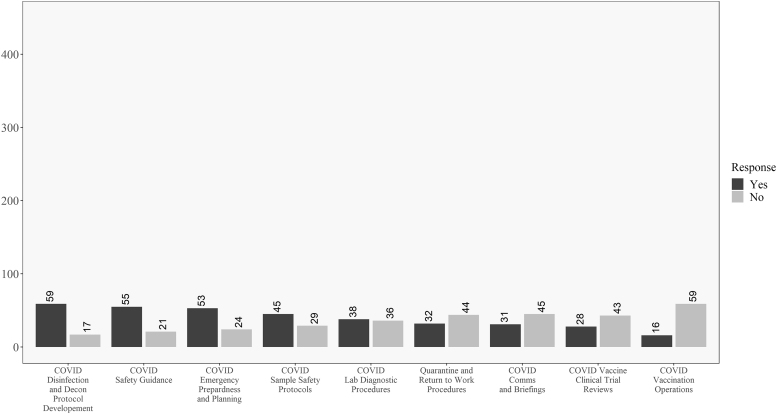
Descriptive results for healthcare respondents on the COVID-19 involvement questions. Questions ordered from highest to lowest of “yes” responses.

Figures 5 and 6 compare academic and government respondents. Working on disinfection and decontamination procedures was relatively common for both. Government workers had the highest observed proportion of “yes” responses to the emergency planning question. Academic workers were relatively more likely to work on vaccine clinical trials. Figures 7 and 8 compare laboratory and healthcare workers. Both groups had almost the same relative ranking of proportions of “yes” responses to the questions.

### Univariate Analysis

Differences by location (United States compared with other countries), sector, and place of employment were explored. Significant differences by location existed in percentage of job involving biosecurity, *p* < 0.001, with U.S. respondents reporting less involvement on average than non-U.S. respondents. Similarly, U.S. respondents (25.9%) reported greater involvement in the review and approval of clinical trials for COVID-19 vaccines than non-U.S. respondents (15.3%), *p* < 0.01, involvement in the development of procedures to analyze or sequence diagnostic or research samples (U.S. 62.8% and non-U.S. 53.6%, *p* < 0.05), and involvement with vaccination logistics, (U.S. 22.3% and non-U.S. 14.0%, *p* < 0.01).

In opposition, more non-U.S. respondents (75.8%) reported working remotely than U.S. respondents (65.5%), *p* < 0.05, and U.S. respondents (42.5%) experienced less involvement in developing quarantine or return-to-work procedures than non-U.S. respondents 53.6%, *p* < 0.05.

Significant differences by sector were also discovered. The percentage of job involving biosecurity resulted in other respondents reporting a higher percentage whose job does not involve biosecurity (14.3%) compared with government employees (2.0%), *p* < 0.05. Similarly, respondents who selected other for their sector reported the lowest amount of workload change “neither increased nor decreased” (23.8%) compared with academic (57.2%), government (61.2%), and nonprofit (78.9%) respondents who reported great increases in workload, *p* < 0.01.

Involvement in COVID-19 response efforts significantly differed, *p* < 0.001, for academic respondents who reported 50.3% involvement in the workplace and community compared with 72.7% of commercial respondents, commercial respondents also differed significantly compared other respondents (30.0%) in workplace and community involvement, and academic respondents involved solely in the community (28.8%) differed from commercial respondents at 7.6%. Involvement in sourcing or vetting PPE, etc. also differed significantly, *p* < 0.05, with commercial respondents involved predominantly in disinfectant and safety supplies (71.2%) compared with academic (44.8%), government (47.9%), and other respondents (28.6%).

In addition, other respondents reported no involvement in sourcing or vetting PPE (52.4%) compared with commercial (16.7%), government (23.3%), and nonprofit respondents (13.2%). Commercial respondents (82.0%) worked remotely compared with nonprofit respondents (50.0%), *p* < 0.05. Involvement in emergency preparation and decision making, *p* < 0.001, occurred at 54.4% for academic participants and 77.2% for government participants. Involvement in the implementation of laboratory diagnostic capabilities, *p* < 0.001, significantly differed for commercial participants (35.6%) compared with government respondents (60.8%).

Academic respondents (31.6%) were also more involved in the review and approval of clinical trials for COVID-19 vaccines, *p* < 0.001, compared with 90.1% of government respondents who were not involved in the review. Significant differences were also found for involvement in the development of procedures to analyze or sequence diagnostic or research samples, *p* < 0.01, with 45.8% of commercial respondents involved compared with 78.4% of nonprofit respondents, as well as nonprofit respondents (78.4%) significantly differing from the 36.8% respondents involved from the other sector.

Involvement in developing quarantine or return-to-work procedures, *p* < 0.05, occurred for 40.6% of academic respondents compared with 64.6% of commercial respondents. And 54.4% of commercial respondents participated in the development of procedures for disinfection and decontamination compared with 69.7% of academic respondents, *p* < 0.05. Finally, involvement with COVID-19 communications or press briefings, *p* < 0.01, differed for 73.3% of academic participants not involved in communications or press briefings compared with 54.1% of nonprofit participants and 41.4% of commercial participants who were involved.

Finally, differences were explored by place of employment. Significant differences were found for workload increase, *p* < 0.001 with regard to several responses. For respondents who selected neither increased or decreased, differences existed between consulting (26.2%) and healthcare (2.6%), and between consulting (26.2%) and manufacturing (35.7%). For respondents who selected increased greatly, differences existed between healthcare (71.4%) compared to both consulting (38.1%) and other (41.1%), as well as between laboratory (63.7%) compared with both consulting (38.1%) and other (41.1%).

Involvement in response efforts significantly differed by place of employment, *p* < 0.001, with differences between consulting (7.2%) respondents compared with laboratory (0.9%) respondents working with clients, consulting (4.9%) respondents compared with manufacturing (28.6%) respondents, and consulting (4.9%) respondents compared with other (20.2%) respondents working with the workplace, pharmaceutical respondents (80.0%) compared with consulting (39.0%) respondents working with workplace and community, and consulting (14.6%) respondents compared with healthcare (14.6%) respondents and laboratory (1.6%) respondents working with community and international groups.

Place of employment differences occurred with regard to involvement in emergency preparation and decision making, *p* < 0.01, such that 66.9% laboratory respondents were involved compared with 52.9% who did not do this study in other places of employment. Involvement in the implementation of laboratory diagnostic capabilities, *p* < 0.01, occurred at 59.9% for laboratory respondents compared with 39.0% for other respondents. Involvement in the review and approval of clinical trials for COVID-19 vaccines, *p* < 0.05, differed for healthcare (39.4%) and laboratory respondents (21.1%) as well as healthcare (39.4%) and other workers (17.3%).

Involvement in the development of procedures to analyze or sequence diagnostic or research samples, *p* < 0.001, existed with 67.9% of laboratory respondents compared with 44.6% of other respondents. Then, 81.0% of consulting respondents were involved in developing safety guidance or procedures compared with 35.7% of manufacturing respondents, *p* < 0.01.

Involvement in developing quarantine or return-to-work procedures, *p* < 0.01, differed for 85.0% of pharmaceutical respondents compared with 42.1% healthcare, 43.4% laboratory, and 46.0% other respondents. And 75.2% of laboratory respondents were involved in the development of procedures for disinfection and decontamination compared with 58.6% other respondents, *p* < 0.01. Finally, involvement with COVID-19 communications or press briefings, *p* < 0.05, differed with 63.2% of pharmaceutical respondents compared with 26.7% other respondents.

As already discussed, differences between the United States and other countries were fewer compared with differences between sector and industry (Table 3). In addition, Table 3 displays how sector was the only variable where significant differences existed for involvement in sourcing or vetting appropriate PPE and workload change. Similarly, place of employment was the only variable where significant differences existed for involvement in developing safety guidance or procedures for healthcare workers, first responders, or the general population. Finally, location was the only variable that differed for the question regarding involvement in vaccination logistics and operations.

**Table 3.** Univariate differences compared by sector, location, and place of employment

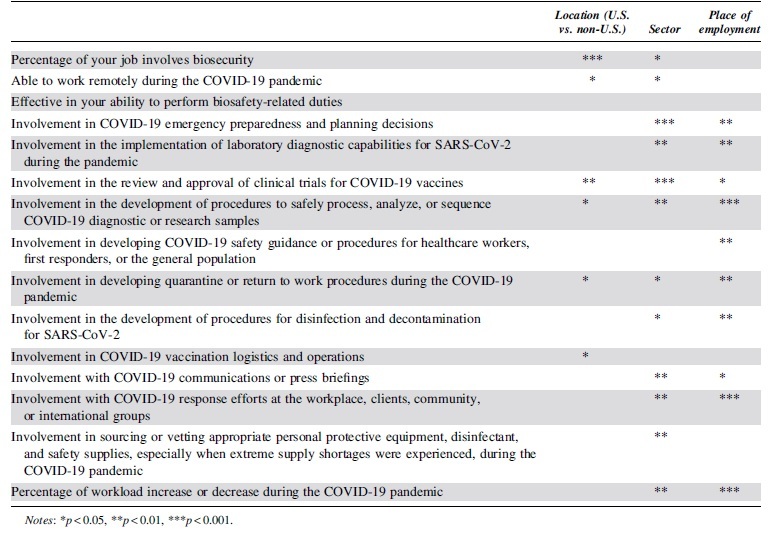

Discrepancies between the raw numbers in Figures 3 and 4 and the univariate analysis are due to including nonresponders on the location question in the “Non-U.S.” group in the table. No differences existed for the question related to personal belief in effectiveness in ability to perform biosafety-related duties such that most participants believed they were effective in their jobs during COVID-19.

### Predictive Analytics

**Figure 9.**
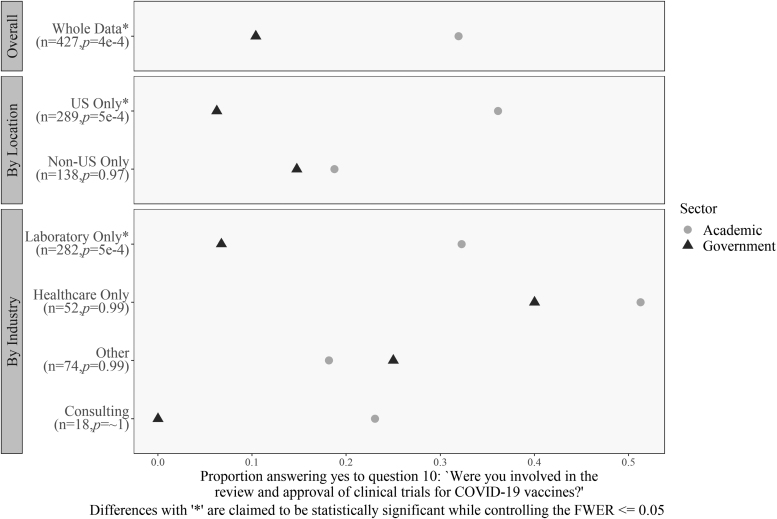
Differences in reviewing vaccine clinical trials by location, industry, and sector. For U.S. respondents and for laboratory workers, academic workers were significantly more likely than government workers to work on COVID-19 vaccine trials. Differences with “*” are claimed to be statistically significant while controlling the FWER ≤0.05.

Several multivariate results need to be reported. The first result is that academic respondents from the United States and those who work in a laboratory setting were more likely than those similarly situated in government employment to answer “yes” to the question, “Were you involved in the review and approval of clinical trials for COVID-19 vaccines?” Figure 9 displays the findings for differences on the question of whether respondents were likely to review clinical trials and research. Overall, academic respondents were more likely to review clinical trials for the vaccine; however, there were significant differences in this trend due to location and industry. For both in and outside the United States, academic respondents were more likely to say “yes”—but the magnitude of the difference is much larger in the United States (OR = 11.1 compared with OR = 1.3).

The gap between academic respondents and government laboratory workers was also large (OR = 8.3). Finally, the contrast between academic and government laboratory workers in the United States was large (OR = 32.3), but we could not satisfactorily determine statistical significance for the three-way interaction. Thus, there is a significant difference between respondents in academic and government laboratories.

**Figure 10.**
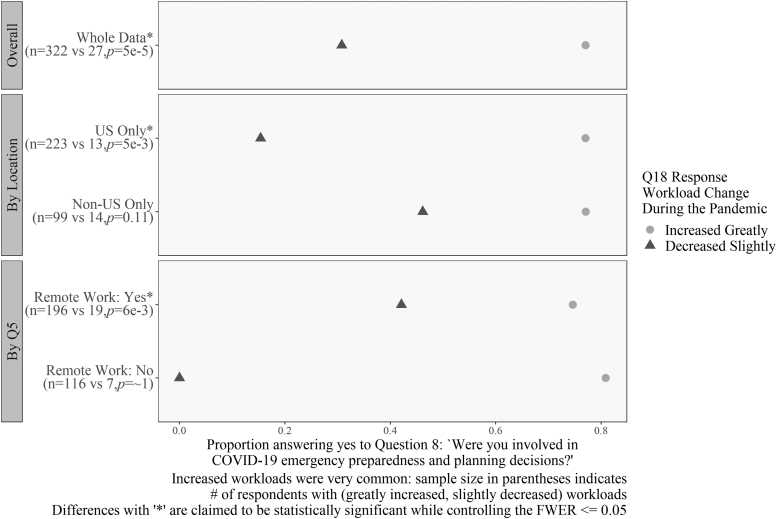
Differences in workload for emergency preparedness and planning decisions. Workload decreased as respondents answered “no” to Question 8 for respondents in the United States and remote workers. Sample size in parentheses indicates the number of respondents with greatly increased and slightly decreased workloads. Differences with “*” are claimed to be statistically significant while controlling the FWER ≤0.05.

**Figure 11.**
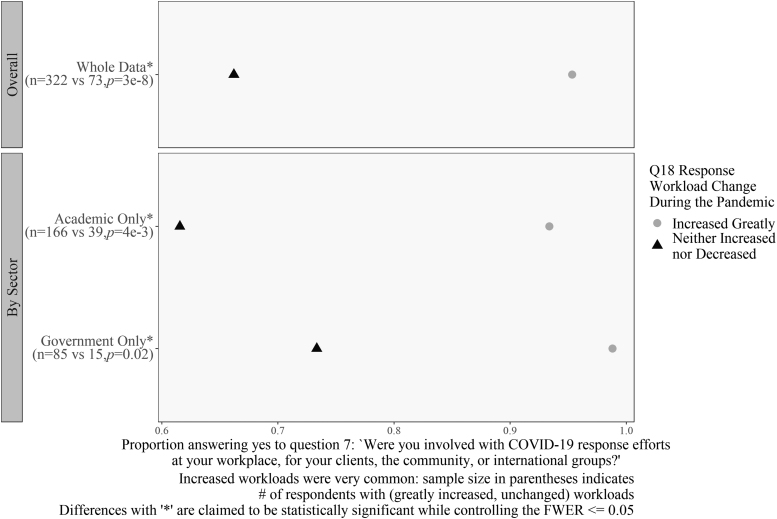
Differences in workload for COVID-19 response efforts. Workload decreased as respondents answered “no” to Question 7 for respondents in academia and government. Sample size in parentheses indicates the number of respondents with greatly increased and unchanged workloads. Differences with “*” are claimed to be statistically significant while controlling the FWER ≤0.05.

**Figure 12.**
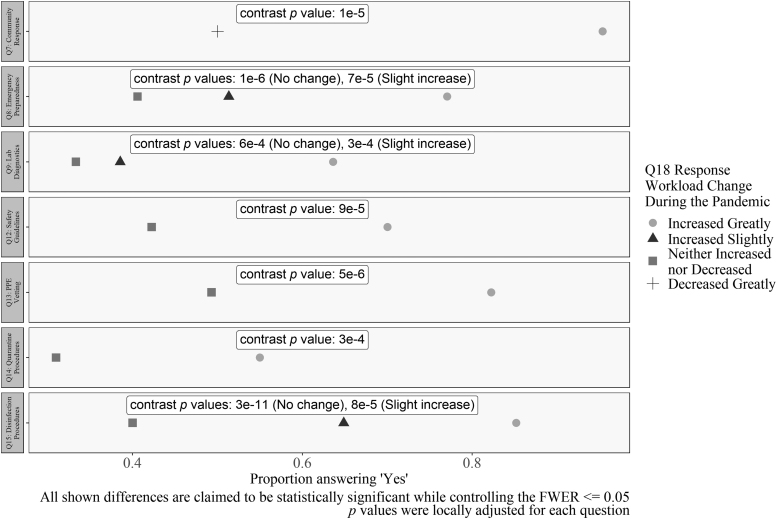
Differences in workload and COVID-19 involvement. Workload decreased when respondents answered “no” to Questions 7, 8, 9, 12, 13, 14, and 15. All *p* values were locally adjusted for each question. All shown differences are claimed to be statistically significant while controlling the FWER ≤0.05.

Figures 10–12 display the results related to questions associated with a change in self-reported workload. Figures 10 and 11, specifically, are associated with interaction patterns between workload and other covariates. Specifically, Figure 10 compares responses to the question “Were you involved in emergency preparedness?” with two levels of the workload question, as well as by location and remote work status.

Analysis revealed that respondents whose workload increased greatly (59% of the total) were more likely to have done some contingency planning than those whose workload decreased slightly (5% of the total) (OR = 8.9). The sample size numbers indicate the thinness of the “decrease slightly” group, but the differences are so large that we can establish a significant difference for U.S. respondents (OR = 40) and those who were able to work remotely (OR = 5.7).

Figure 11 shows a similar comparison for the question, “Were you involved in COVID-19 response efforts in your community?” Respondents whose work increased greatly were significantly more likely than those whose workload did not change (13% of the total) to say that they had done work to support their community or workplace. This pattern held for both academic and government respondents (OR = 7 and OR = 58.8, respectively).

The final group contains general findings, not specific interactions as mentioned. Respondents who reported that their workload increased greatly were more likely to answer “yes” to certain questions as noted hereunder. For each question hereunder, the largest OR between workload options with a significant difference in the proportion of “yes” responses (e.g., Increased Greatly vs. Decreased Greatly) is reported (Figure 12).

The questions where a “yes” response was correlated with an increased workload were: The involvement in community response (OR = 32.3); involvement in emergency preparedness (OR = 5); involvement in the implementation of laboratory diagnostic capabilities for SARS-CoV-2 (OR = 3.4); involvement in developing COVID-19 safety guidance or procedures (OR = 3.9); involvement in sourcing or vetting appropriate PPE, disinfectant, and safety supplies (OR = 4.5); involvement with developing quarantine or return-to-work procedures (OR = 3.7); and involvement in the development of procedures for disinfection and decontamination for SARS-CoV-2 (OR = 9.1).

In contrast, this indicates that significant reductions in workload were recorded for respondents who selected “no” for the mentioned questions. The “increased greatly” category was always the baseline. For example, for the question “Were you involved in the implementation of laboratory diagnostic capabilities?” both the “increased slightly” and “no change” group were significantly more likely to answer “no” than those whose work increased greatly. If greatly increased workload decreased the respondents' quality of life, this means that implementing safety procedures for COVID-19 diagnostic laboratories was associated with increases in stress. A causal relationship is possible but was not formally tested by the current methods.

### Qualitative Results

From the analysis of the open response questions, three major themes emerged: work, home, and a combination of challenges that affected both work and home. Challenges that respondents felt affected work fell into five categories: biosafety protocols, decision making, biosafety-related duties, physical work environment, and job satisfaction. Challenges that affected home fell into one primary category: personal well-being. Finally, some challenges affected both work and home life.

#### Work

Work-related concerns during the pandemic covered changes to how high-level decisions were made, the actual work done by biosafety professionals, the logistical situation their laboratories faced, and employee satisfaction. The overall tone of the comments was negative. The pandemic created uncertainty around laboratory protocols, increased the workload of biosafety professionals, undermined their expertise, and generally made the workplace challenging. Comments ranged from being more matter of fact to showing serious frustration.

#### Biosafety protocols

In prepandemic conditions, biosafety professionals were expected to provide comprehensive yet actionable guidance about laboratory safety. The difficulty of this task increased during the pandemic. One respondent was “expected to have a solution or a response to every COVID-19 related situations [sic].” The changing situation on the ground made communication and implementation of protocols confusing: “Implementing the ever changing and at times contradicting guidance from government. Ensuring employees and patients understand the requirements and working to ensure the majority accepted the changes…”

Biosafety professionals also struggled to accommodate the interdisciplinary demand for research opportunities: “With the influx of suddenly available funding for COVID-19 related sampling, testing, etc., I found that investigators with no previous experience handling infectious materials or pathogens were getting funding and embarking on projects they were woefully unqualified and untrained for. We have been lucky that as far as we know, no workers have become infected from these activities.” Biosafety professionals had to adjust their expectations for the scope, stability, and user experience level of their guidance.

#### Decision making

Biosafety respondents felt their influence within their workplace declined during the pandemic, thereby making it more difficult for them to recommend and advance safety protocols. Leadership was often sensitive to politics as well as resistance to new recommendations. As one frustrated respondent put it: “VERY DIFFICULTLY [sic] CONVINCED PEOPLE TO ADOPT SAFETY PARAMETERS DURING COVID.” Media and society interfered with protocol acceptance: “The politics got in the way of conveying the science and safety suffered. The people initially conveying the message were not trained in how to handle this kind of crisis specifically. It was hard to watch.”

Another respondent felt the public discourse around COVID-19 diminished their work: “To keep a positive attitude while media (news outlets and social media) minimized the public health actions we were trying to implement.” Sometimes organizational structure reduced the influence of biosafety professionals: “My institution has one of the largest biosafety programs in the country, and yet the expertise of our office was often overlooked. This may be a consequence of our biosafety program being in a separate division from Environmental Health and Safety. I think that sometimes people conflate our EH&S program with our biosafety program.” Implementation of biosafety guidance became more difficult for interpersonal, social, and organizational reasons.

#### Biosafety officer duties

During the pandemic, biosafety professionals faced challenges with their day-to-day work. There were oft-cited problems around the management of the laboratory's workforce. One respondent described their challenges as “Continuance of workflow when critical personnel needed to intermittently miss work/quarantine/isolate selves or family members. Highly skilled or specifically skilled personnel suddenly missing work played heavily in providing continued performance of research and in workload distributions during the high-load periods or critical study phases.”

Productivity suffered: “Our site was at 25% capacity, and I worked from home nearly 100% of the time. Safety is not done effectively from behind a desk.” Another respondent was blunter: “staff is unproductive and lazy in the home office.” Besides training and management, termination of employees puts a strain on biosafety professionals: “Having to dismiss critical employees refusing vaccinations.” Workplace management deteriorated during the pandemic.

#### Physical work environment

Besides managing employees, biosafety professionals were responsible for finding materials and maintaining the safety of laboratories. Logistical challenges were a common theme—one respondent mentioned, “Sourcing PPE and disinfectants. Finding space to spread out our researchers in our cramped tissue culture facilities.” Employee PPE was also critical. Arranging well-fitting equipment and training employees was not easy: “Fit testing and training of employees and students was a massive challenge, and biosafety staff were required to help with this effort since more than 4X the normal number of individuals needed respirator fit testing and training.” This set of issues placed additional time and effort challenges on biosafety professionals during the pandemic.

#### Job satisfaction

During the pandemic, overall job satisfaction decreased while stress increased. A typical description of the situation was: “We were and still are short-staffed. We have been unable to hire and retain competent individuals and have asked them to work under extremely difficult conditions. We have also been dealing with staff burnout and low morale. Several of these issues stem from a lack of support and understanding from the administration about the challenges staff have been facing.” Biosafety officers reported their efforts were frequently overturned when guidance or policies changed at the governmental and/or institutional level. Another respondent mentioned, “Keeping essential personnel engaged and feeling appreciated.” Recruitment and retention issues among biosafety professionals were consistent problems during the pandemic.

#### Home life

Challenges to well-being also came up with respect to the respondents' home life during the pandemic. Some respondents brought up caring for family members in addition to their work duties: “Balancing personal responsibilities with an extremely heavy and urgent workload and no breaks for respite (as a parent with young children without COVID-safe caregivers).” Some emotional difficulties struck deep: respondents mentioned feeling “existential dread” and “fighting stigma and hopelessness.” Opportunities to recover also diminished: “I was also checking e-mail into evening, weekend, and on vacation and still are (sic).” These circumstances seemed to add more stress to the lives of biosafety professionals during the pandemic.

#### Work and home life

Some themes intersected the respondents' work and family responsibilities. For example, lack of clear protocols for COVID-19 increased one respondent's stress about spreading the disease: “Because initially there was alot [sic] of confusion and not enough proven answers to how safe we were working in the lab daily with fellow workers. I was VERY worried every day that by going to work, I was going to be exposed and bring home COVID to my spouse, who was considered at risk. But I was repeatedly reminded that my presence at work was required.”

There were financial costs to biosafety professionals from setting up their home office: “Getting used to not having the same resources at home, as I did at work. (e.g., Access to laser printers and paying for printer cartridges).” One respondent's comment could apply to both areas; they felt that their role was about, “Balancing others out—not letting them minimze [sic] or over-react.” During the pandemic, biosafety professionals were called to deliver more guidance and to manage creatively and maintain a safe work environment, while working with leadership that did not always value their advice. The results of this were diminished effectiveness and negative consequences for their families and their own overall happiness.

## Discussion

Descriptively, there are some interesting findings related to U.S. and non-U.S. responses to COVID-19. Most respondents were involved in traditional biosafety duties^30^ during the pandemic, with the exception of the level of involvement in creating return-to-work and quarantine procedures. As shown in Figures 3 and 4, one difference worth highlighting is that non-U.S. respondents were more engaged in return-to-work and quarantine efforts as well as communications and briefings, but less involved in vaccine clinical trial reviews and vaccination efforts than their U.S.-based counterparts.

The univariate analysis revealed non-significant differences in workload increase for U.S. and non-U.S. respondents with U.S. respondents reporting higher levels of workload increase. Additional significant differences existed in working remotely, involvement in the review and approval of clinical trials, involvement in the development of procedures to safely process or analyze research samples, involvement in developing quarantine and return-to-work procedures, and involvement in vaccination logistics. Both sector and place of employment seemed to be a determining factor with significant differences existing for most questions. Although significant differences existed for all three variables, sector resulted in the most differences, followed by place of employment, and then finally whether someone was in the United States or not.

The multivariate analysis showed that academic respondents who lived in the United States and academic respondents who worked in a laboratory setting were more likely than those who worked for the government to respond “yes” to questions regarding review and approval of clinical trials for vaccines. This may be due to many academic institutions having local oversight mechanisms (e.g., IBCs and IRBs), pre-existing relationships with hospitals and medical centers, and the accelerated and collective nature of the COVID-19 vaccine development and approval process.

There was a greater need or interest in the United States to utilize academic research resources to clinically assess vaccines. Similarly, the interaction effects in the workload questions align with expectations. Biosafety professionals whose workload increased were generally tasked with a wider variety of duties than those whose workload did not increase.

Similar to other frontline workers involved with the COVID-19 pandemic response,^31^ biosafety professionals also saw their mental health and well-being suffer. This finding aligns with our second multivariate result already presented: burnout and unclear responsibilities were common experiences for the respondents. Descriptive statistics show how biosafety professionals had varying degrees of responsibility in the pandemic response, with some international differences. The most telling responses were those from the qualitative analysis and the challenges faced when working from home or at the office, which included the difficulty in finding a good work/home balance, stress from the constant influx of health and safety guidance, and the inability to find respite.

Our findings suggest that evidence-based measures could be developed that would better support biosafety officers when responding to an emergency. A general recommendation from this study is to look for answers in the minority of cases where things went right, rather than in the majority where the job became more difficult. This would help answer the question “what does good look like?” There was a consistent multivariate trend that involvement in various pandemic tasks was associated with more workload. Therefore, there is a plausible connection between increased workload and higher stress.

Learning more about this connection means asking follow-up questions for future research, such as: Which combinations of pandemic-related tasks did an individual perform, and what could be said about the structure of their organizations? Did organizations have less or more redundancy in the number of biosafety positions? Did biosafety professionals have greater or lesser institutional authority prepandemic? Did biosafety professionals work in a region with more or less caseloads? Similarly, there appear to be interaction effects occurring between the key variables—sector, industry, and location.

As such, more statistical analysis is necessary to parse out the interaction effect that may be occurring between these key elements. Answering these questions and conducting additional analyses will be helpful to calibrating the workload of biosafety professionals in the future. Biosafety expertise is needed but asking them to do too much is likely unsustainable.

A second direction of future research is to look more closely at location differences. For this study, the location variable was dichotomized into United States/non-United States since most respondents were in the United States. This was not the only way to look at location. Other alternatives could have been to compare the Global North and Global South, or to break the United States into geographical or political regions. For example, were the academic laboratory workers in the United States, who were observed to be much more likely than their government counterparts to review vaccine trials, evenly spread around the country? Or were they clustered in a few centers? These questions could not be answered with high reliability within this study, but future research could reveal institutional or funding differences between locations.

## Conclusion

In this article, an analysis of several factors about the biosafety response to the COVID-19 pandemic was presented. This analysis will help inform and better prepare the biosafety community and others in future pandemics. Additional surveys of international respondents, as well as future longitudinal studies, could better inform how biosafety professionals reacted more broadly to the pandemic response over time. Biosafety professionals were likely to experience increased stress and that the modal respondent's workload increased greatly. There might have been more differences in behavior between employment sectors, workplace, and location, but only one multivariate sector difference could be detected with high reliability.

There were some limitations to the study and analysis that future studies could improve upon. First, the main thing that survey would be to increase the sample size outside the United States. A wider pool of responses from a variety of non-U.S. countries could allow for direct analysis between countries or a wider investigation on differences between the experiences of professionals in the Global North versus the Global South. In addition, future studies could go in an exploratory or confirmatory direction. A deeper exploratory study could find interactions between sector, location, and industry that predicted behavior. Some clustering analysis was done on the present data, but more could have been done to find better clusters and to validate them.

A focused confirmatory analysis could take the main findings and subject them to a specific survey and comparison. Although the multivariate inferential findings here are claimed to be tested with high severity, this approach was exploratory in nature. A set of statistical models was run against all the questions—the project did not have a specific statistical comparison in mind at the start. Further attempts to confirm or refute our findings with focused surveys of biosafety professionals would be worthwhile.

## Supplementary Material

Supplemental data

Supplemental data
