# Supplementary material for: Experiences During the COVID-19 Pandemic: A Survey of Biosafety Professionals
Source: Appl Biosaf. 2022 Sep 14;27(3):127–43. doi: 10.1089/apb.2022.0012 (PMC9526473; doi:10.1089/apb.2022.0012)
Supplement: Supplemental data [file Supp_DataS1.docx]

**Supplement A – Institutional Review Board (IRB) Approved Survey Questions**

Q1. **Do you agree to be a part of the study**? oYes

Q2 **Are you 18 years of age or older?**

oYes, I am 18 years of age or older

oNo, I am under 18 years of age

Q3 **Are you currently employed in a position with biosafety responsibilities?**

oYes
oNo

Q4 **What percentage of your job involves biosecurity?**

o76-100%

o 51-75%

o 26-50%

o 1-25%

o My job does not involve biosecurity

Q5 **Were you able to work remotely while performing biosafety related duties during the COVID-19 pandemic?**

oYes
oNo
oNot Applicable

Q6 **Do you believe that you were effective in your ability to perform biosafety related duties?**

oYes
oNo

oNot Applicable

Q7 **Were you involved with COVID-19 response efforts at your workplace, for your clients, the community** (schools, nursing homes, religious community, city or town, etc.) **or international groups?** Please select all that apply.

▢ Yes, for my workplace
▢ Yes, for clients
▢ Yes, for the community
▢ Yes, for international groups
▢ No, I was not involved in these activities

▢ Not Applicable

Q8 **Were you involved in COVID-19 emergency preparedness and planning decisions?** oYes

oNo
oNot Applicable

Q9 **Were you involved in the implementation of laboratory diagnostic capabilities for SARS-CoV-2 during the pandemic?**

oYes
oNo
oNot Applicable

Q10 **Were you involved in the review and approval of clinical trials for COVID-19 vaccines?**

oYes
oNo
oNot Applicable

Q11 **Were you involved in the development of procedures to safely process, analyze or sequence COVID-19 diagnostic or research samples?**

oYes
oNo
oNot Applicable

Q12 **Were you involved in developing COVID-19 safety guidance or procedures for healthcare workers, first responders, or the general population?**

oYes
oNo
oNot Applicable

Q13 **Were you involved in sourcing or vetting appropriate personal protective equipment, disinfectant, and safety supplies, especially when extreme supply shortages were experienced, during the COVID-19 pandemic?**

▢ Yes, personal protective equipment

▢Yes,disinfectant
▢ Yes, safety supplies
▢ No, I was not involved in this activity

▢ Not applicable

Q14 **Were you involved with developing quarantine or return to work procedures during the COVID-19 pandemic?**

oYes
oNo
oNot Applicable

Q15 **Were you involved in the development of procedures for disinfection and decontamination for SARS-CoV-2?**

oYes
oNo
oNot Applicable

Q16 **Were you involved in COVID-19 vaccination logistics and operations?**

oYes

oNo
oNot Applicable

Q17 **Were you involved with COVID-19 communications or press briefings?**

oYes

oNo
oNot Applicable

Q18 **How much did your workload increase or decrease during the COVID-19 pandemic?** oIncreased greatly

oIncreased slightly
oNeither increased or decreased

oDecreased slightly

oDecreased greatly (5)

Q19 **What were your greatest challenges in the workplace during the pandemic?**

Q20 **Do you have any other comments about your involvement during the COVID-19 pandemic that is above and beyond the questions asked within this survey?**

Q21 **Which sector best describes your place of employment?**

oAcademic
oCommercial
oGovernment

oNon-Profit

oOther

Q22 **Which industry function best describes your place of employment?**

oConsulting
oHealthcare
oLaboratory

oManufacturing

oPharmaceutical

oOther

Q23 **For which country do you currently perform the most of your work?**

Q24 **ABSA International's Journal of *Applied Biosafety* is planning to publish a special themed issue about the COVID-19 pandemic. Are you interested in writing about your biosafety experiences during the pandemic, either independently or as part of a collaborative project?**

oYes

oNo

Q25 **Which COVID-19 biosafety topic(s) are you most interested in writing about?**
