# Supplementary material for: Experiences During the COVID-19 Pandemic: A Survey of Biosafety Professionals
Source: Appl Biosaf. 2022 Sep 14;27(3):127–43. doi: 10.1089/apb.2022.0012 (PMC9526473; doi:10.1089/apb.2022.0012)

# **Supplement B – Statistical Analysis Discussion**

# **Significant Findings Justification**

Consider a claim that did not make it into the main section of the paper. For the question: “were you involved in COVID-19 emergency preparedness and planning?”, respondents in government were more likely than those in academia to say yes (second table). The odds ratio, adjusting for the other variables in the model, is 2.5. The *p* value for the contrast, after adjusting for multiple comparisons, is 0.005. Outside of the context of the data analysis as a whole, this is a plausible and statistically impressive finding.

Nevertheless, we did not include it amongst our significant multivariate findings. This is because a *p* value threshold of 0.005 is not a highly reliable threshold for our data set and analysis strategy. Although we did not preregister the analysis, we examined the reliability of our strategy. How often would it report false findings, assuming that our response and explanatory variables were totally unrelated? A *p* value threshold of 0.005 does not quite get us to a highly reliable method – we found that level of reliability at 0.001.

Figure 1 in the paper shows the output of this examination, and the following is an explanation of its results. The results are based on a null hypothesis simulation. This means that we took all the binary response columns, and randomly re-shuffled their values. The columns with explanatory variables were kept the same. Any theoretical association between the response and predictors should be broken. However, the simulated data is still relevant to the actual problem – we have the same proportion of respondents in academia, government and so on. This is an example of a data guided simulation.^1^

We ran this simulation 200 times. For each simulation run, we re-ran our entire multivariate analysis. This comes out to 609 pairwise comparisons across all the responses and all the explanatory variables. We kept track of the effect sizes and *p* values from these contrasts, just as with the real data. We could then see how many times we observed a *p* value below a given threshold.

The columns in the Figure 1 correspond to *p* value thresholds – this means reporting any contrast with a *p* value below 0.05, 0.01, 0.005 or 0.001 as statistically significant. The rows represent outcomes – how many false positive claims were observed. The bottom row is the ideal scenario – “no false positives.” Outcomes get worse as the vertical axis increases, and the shade of the tile indicates how often (out of 200 simulations) these outcomes occurred.

With a threshold of 0.05 (adjusted for multiple comparisons within a particular response/explanatory variable), it was uncommon to have zero false positives. The modal scenario was 3-5 false positives, and about 20% of the time at least 6 were reported. If we were to take 0.05 as our threshold, we would have a roughly 20% chance of claiming that around 1% of the potential contrasts were non-zero, even if we knew that there was no true difference in response outcomes by any of the explanatory variables.

This was not the desired outcome – we want to control the family wise error rate (FWER), which is 1 minus the bottom row of Figure 1. A FWER close to 0.05 is desired. This is what the Tukey HSD adjustment provides locally.^2^ For the original motivating example: if we were to just study covid emergency planning, and use the same model, we could have used an adjusted *p* value threshold of 0.05 to maintain a FWER of 0.05. The claim that government respondents were more likely than academics to do emergency planning meets this standard.

If we were to apply a 0.005 threshold for our entire analysis, we would have a FWER of 0.25. This is better than with a threshold of 0.05, but not good enough. We only get close to the desired level with a threshold of 0.001. We therefore have a method of selecting important contrasts with at least some demonstrated reliability.

The decision to not include the government vs academia comparison for emergency response is part of the price we paid for analytical flexibility. We had a general idea of using location, sector, industry, job status, workload change and remote work status as variables, but did not start with a prespecified set of questions. We wanted to cast as wide of a net as possible, and then account for this search to control the probability of reporting false positives. Had we started with a narrower, preregistered plan, we might well have declared the above comparison to be significant.

## **Interaction Effects**

The same type of analysis is behind the claim of statistically significant interaction contrasts for questions 10 (vaccine review), 8 (emergency preparedness) and 7 (community response). Consider our finding that academics in the US were more likely than government workers to review the vaccine. We came to this finding through a selective procedure. Since the main effect contrast had a *p* value below our threshold of 0.001, we considered possible interactions.

Importantly, we subsetted the data to just academic or government respondents and searched for interactions with the other covariates. There are now six possible interaction contrasts between location and sector. We were mainly interested in differences between academics and government workers within a location – this corresponds to the contrasts US/Academic – US/Government and Non-US/Academic – Non-US/Government. The former had a large observed difference in proportions, and we obtained a small Tukey adjusted *p* value (6e-4).

The potential problem with this is that it is driven by the selection in the first stage of the analysis. The only reason that we considered this interaction was that the main effect had a *p* value below our threshold. This procedure could potentially increase the FWER.

To check for this possibility, we looked back at the simulation behind Figure 1. We looked up the simulated data sets where there was at least one false positive. We then calculated interaction effects for these contrasts, and checked their Tukey adjusted *p* values. None of these *p* values was below 0.05. Therefore, we are willing to use 0.05 for the interaction effect *p* value as a threshold for significance.

## **Motivation and Use Cases**

Finally, we want to be transparent about our motivations. This analysis is guided by the principle of maintaining high severity.^3^ This means that we although we might have seemingly impressive data behind our claims, in this case a large odds ratio, we want to make sure that we could not have produced this evidence if our claims were wrong. Using numerical thresholds to sort potential claims into “passed with high severity” and “did not pass” is a convenient way to organize the results.

However, there is a great deal of debate in the statistics community on this issue. We generally take the position of Mayo and Hand^4^ and Tunç, Tunç and Lakens^5^ that numerical thresholds contribute to scientific discussion. Not everyone agrees with this. Although we cannot make our raw data publicly available, we can share all the contrasts that might potentially be of interest. One use case of the following tables could be to construct a prior distribution for a future Bayesian analysis of a similar population.

A final issue is that our estimates of effect sizes, through the model adjusted odds ratio, could be affected by the “Winner’s Curse.”^6,7^ The general problem is that when effect sizes are only calculated after a numerical threshold is reached, we can no longer assume that the estimate is unbiased. Some new research^8^ attempts to correct for this, but we consider this to be a unsolved problem at the moment.

**References:**

1. Nan Y, Yang Y. Variable selection diagnostics measures for high-dimensional regression. Journal of Computational and Graphical Statistics. 2014 Jul 3;23(3):636-56.
2. Hothorn T, Bretz F, Westfall P. Simultaneous inference in general parametric models. Biom J 2008 Jun;50(3):346-63; doi: 10.1002/bimj.200810425.
3. Mayo DG. Statistical inference as severe testing. Cambridge: Cambridge University Press; 2018.
4. Mayo DG, Hand D. Statistical significance and its critics: practicing damaging science, or damaging scientific practice?. Synthese. 2022 Jun;200(3):1-33.
5. Tunç DU, Tunç MN, Lakens D. The Epistemic and Pragmatic Function of Dichotomous Claims Based on Statistical Hypothesis Tests.
6. Ioannidis JP. Why most discovered true associations are inflated. Epidemiology. 2008 Sep 1:640-8.
7. Do "underpowered" tests "exaggerate" population effects? (IV) [Internet]. Error Statistics Philosophy. 2022 [cited 2022Jun12]. Available from: https://errorstatistics.com/2022/05/02/do-underpowered-tests-exaggerate-population-effects/#comments.
8. van Zwet EW, Cator EA. The significance filter, the winner's curse and the need to shrink. Statistica Neerlandica. 2021 Nov;75(4):437-52.


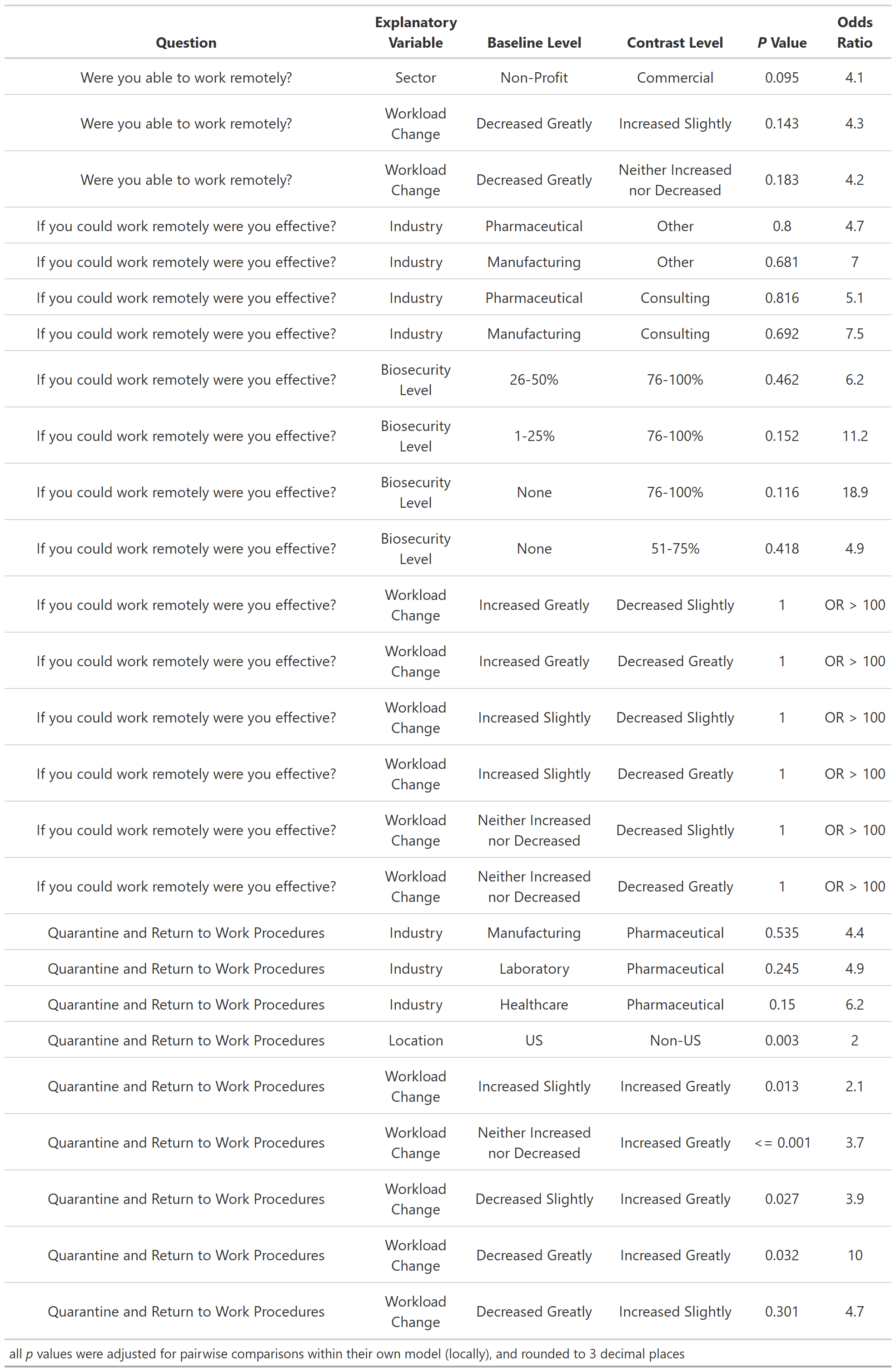


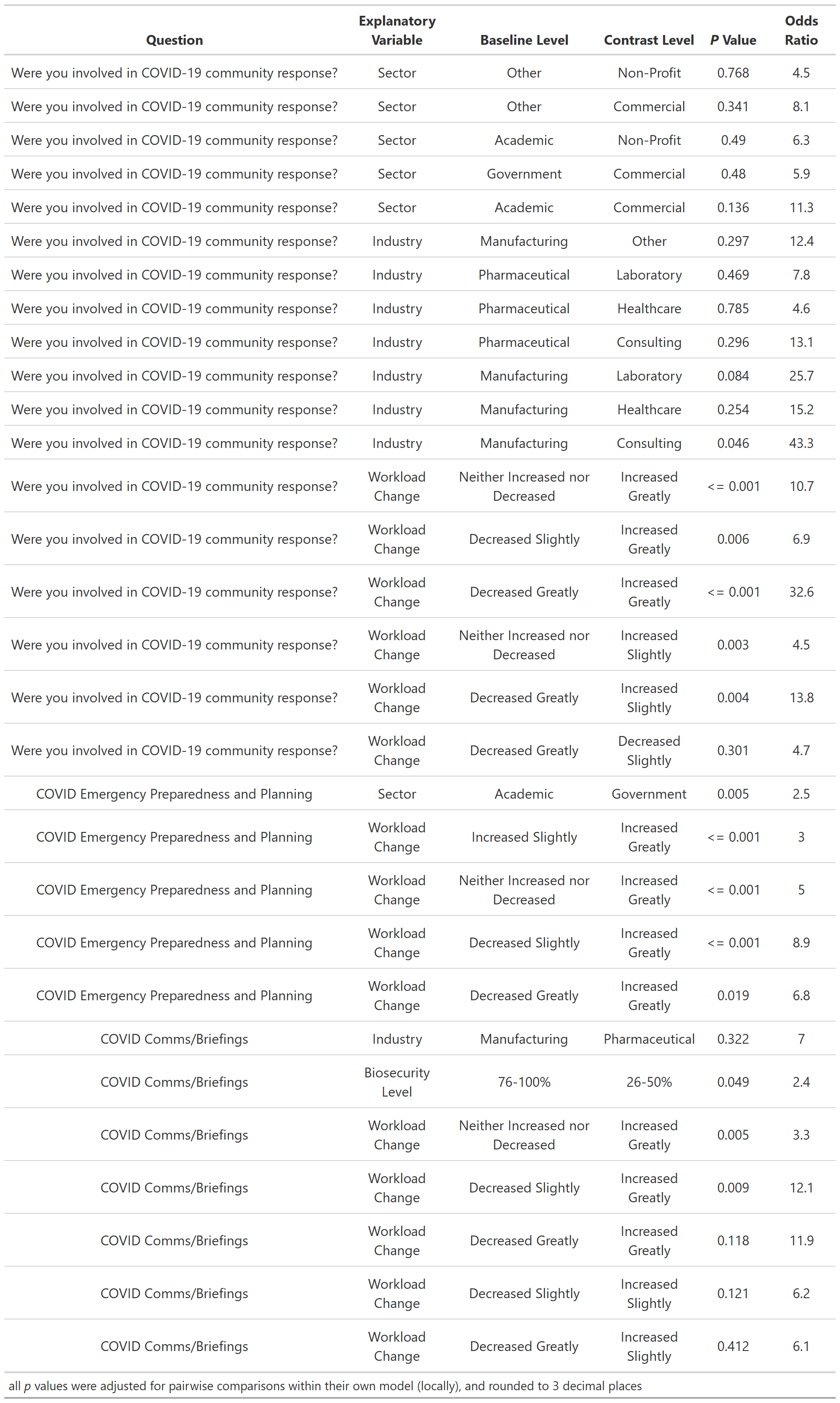


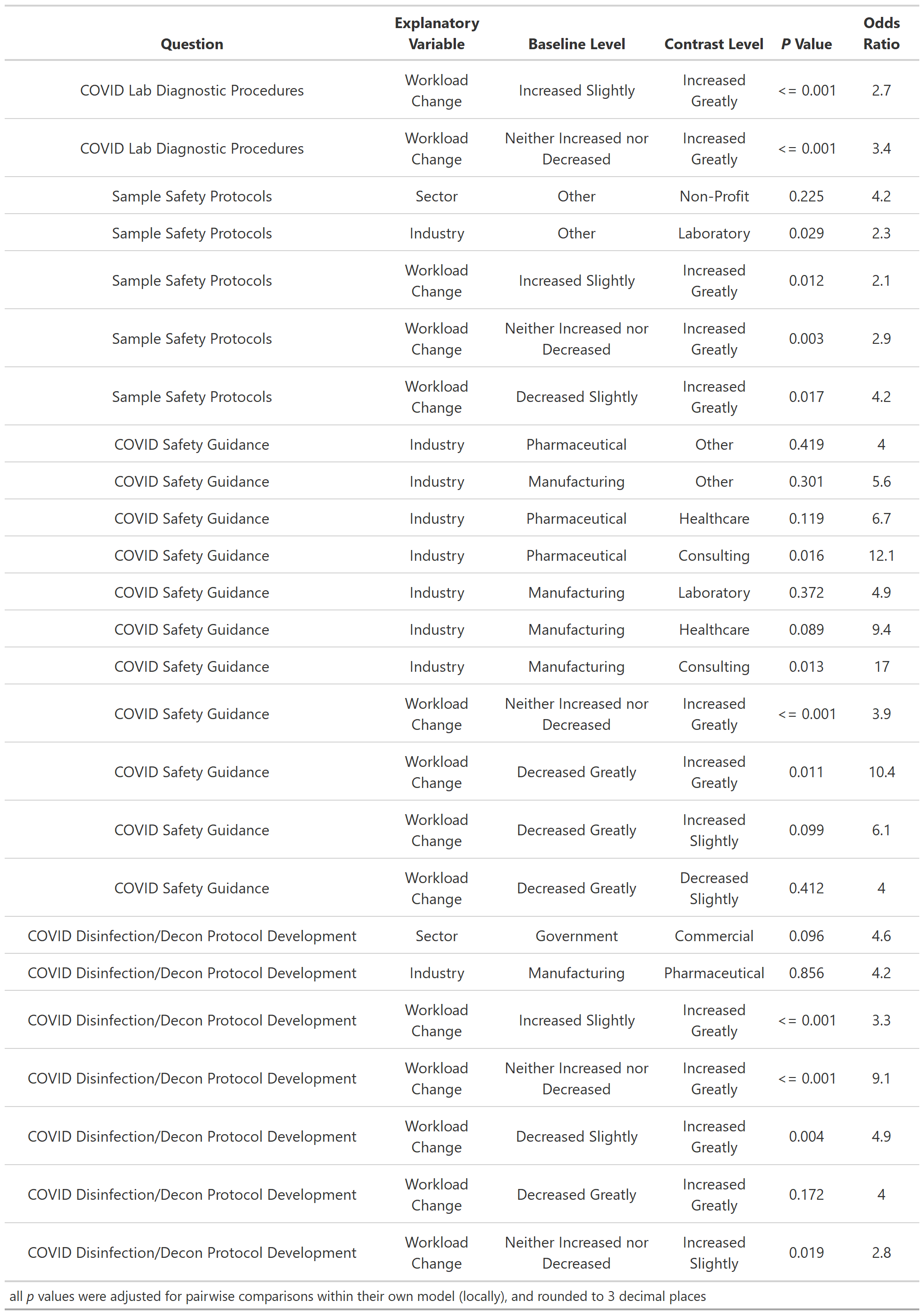


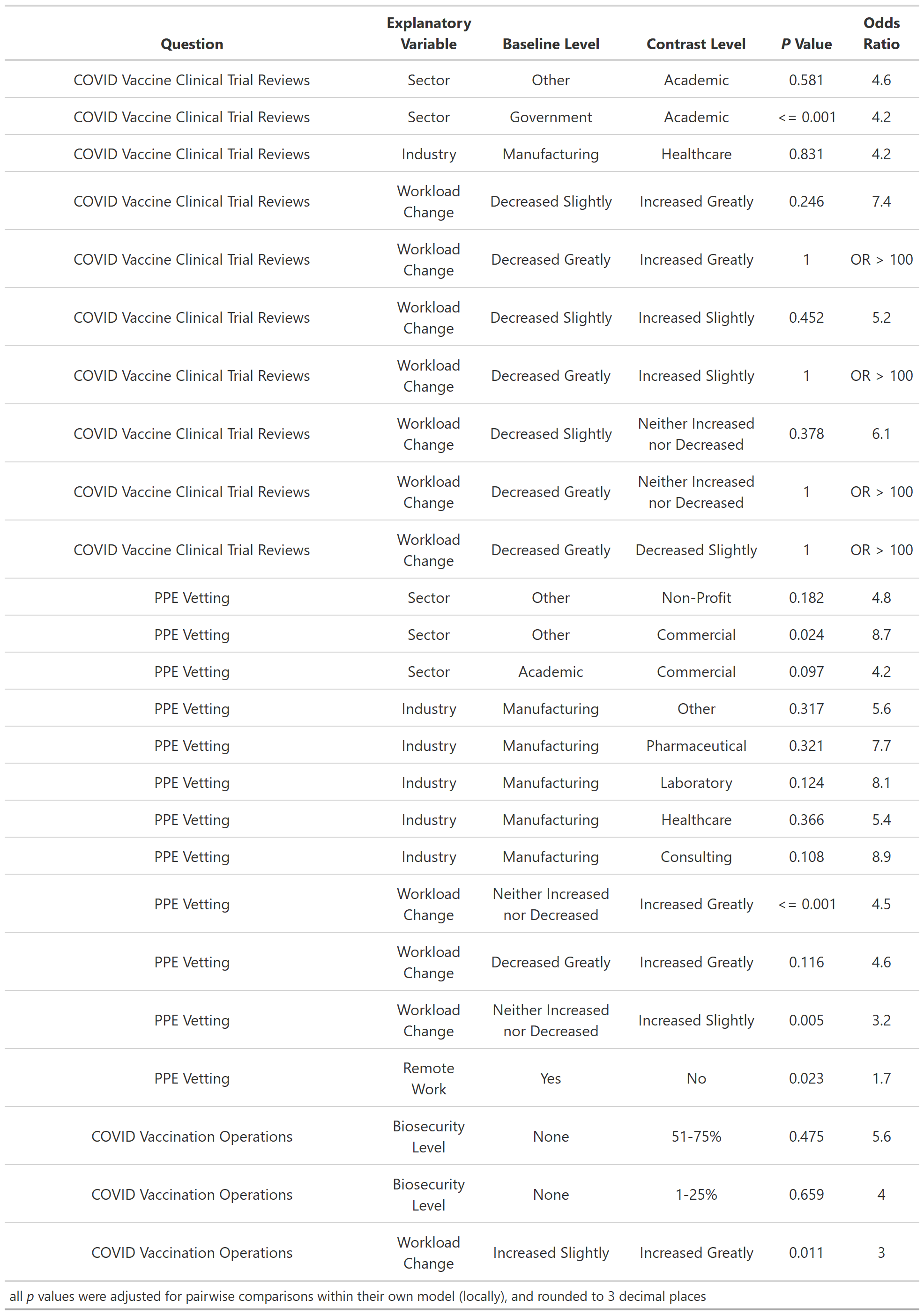

Supplement: Supplemental data [file Supp_DataS2.docx]
